# Transcriptomic Sequencing and Co-Expression Network Analysis on Key Genes and Pathways Regulating Nitrogen Use Efficiency in *Myriophyllum aquaticum*

**DOI:** 10.3390/ijms20071587

**Published:** 2019-03-29

**Authors:** Rui Wang, Shengjun Xu, Cancan Jiang, Haishu Sun, Shugeng Feng, Sining Zhou, Guoqiang Zhuang, Zhihui Bai, Xuliang Zhuang

**Affiliations:** 1 Key Laboratory of Environmental Biotechnology, Research Center for Eco-Environmental Sciences, Chinese Academy of Sciences, Beijing 100085, China; szywangrui@126.com (R.W.); sjxu@rcees.ac.cn (S.X.); yesiamcan@163.com (C.J.); sunhaishu0525@163.com (H.S.); fengshugeng@163.com (S.F.); zsining@hotmail.com (S.Z.); gqzhuang@rcees.ac.cn (G.Z.); zhbai@rcees.ac.cn (Z.B.); 2 College of Resources and Environment, University of Chinese Academy of Sciences, Beijing 100049, China

**Keywords:** nitrogen use efficiency, RNA-sequence, co-expression network analysis, constructed wetlands, nitrogen metabolism, carbohydrate metabolism, transcriptome factors, secondary metabolism

## Abstract

Massively input and accumulated ammonium is one of the main causes of eutrophication in aquatic ecosystems, which severely deteriorates water quality. Previous studies showed that one of the commonly used macrophytes, *Myriophyllum aquaticum*, was capable of not only withstanding ammonium of high concentration, but also efficiently assimilating extracellular ammonium to constitutive amino acids and proteins. However, the genetic mechanism regulating such efficient nitrogen metabolism in *M. aquaticum* is still poorly understood. Therefore, RNA-based analysis was performed in this study to understand the ammonium regulatory mechanism in *M. aquaticum* in response to various concentrations of ammonium. A total of 7721 genes were differentially expressed, of which those related to nitrogen-transport, assimilation, and remobilization were highly-regulated in response to various concentrations of ammonium. We have also identified transcription factors and protein kinases that were rapidly induced in response to ammonium, which suggests their involvement in ammonium-mediated signalling. Meanwhile, secondary metabolism including phenolics and anthocyanins biosynthesis was also activated in response to various concentrations of ammonium, especially at high ammonium concentrations. These results proposed a complex physiological and genetic regulation network related to nitrogen, carbohydrate, transcription factors, and secondary metabolism for nitrogen use efficiency in *M. aquaticum*.

## 1. Introduction

Nitrogen, especially ammonium, is one of the most indispensable nutrients for growth and reproduction of macrophytes in aquatic ecosystems [1,2]. However, dramatically increased ammonium input and accumulation in aquatic ecosystems severely deteriorated the water quality and ecological community as a whole [3,4,5]. Different advanced treatment approaches have been developed and deployed for wastewater treatment. Constructed wetland (CW) is one that has been gaining greater attention in recent years [6]. Due to advantages including easy maintenance, on-site treatment, and good self-purification capacity [6], CWs are widely used to treat wastewater from industrial, agricultural, and livestock breeding activities in many small rural areas where large-scale wastewater treatment plants are scarce [7]. In CWs, macrophytes play important roles in nitrogen removal since macrophytes can absorb and assimilate the nitrogen nutrients to constitute amino acids and proteins in tissues [8,9]. Meanwhile, as economic plant species, many wetlands plant species show rapid growth, produce a large amount of aboveground biomass, and own the potential to be used as a source of feed and biomaterials as well as bioenergy [4,10]. However, ammonium of high concentrations in wastewaters were usually toxic to most macrophytes, which influenced the treatment effects of CWs [4,6].

Generally, plants including macrophytes prefer to uptake ammonium nitrogen since it is less energy-consuming [2,11], and all inorganic nitrogen is first reduced to ammonium, which will be assimilated by plants into nitrogen-carrying amino acids [12,13]. Ammonium in plants such as *Arabidopsis* can originate from nitrate reduction, direct absorption, photorespiration, or deamination of nitrogenous compounds, such as asparagine [2,11]. Afterward, ammonium enters the biosynthetic pathways of plant cells in order to produce different amino acids [2], which are used to synthesize proteins and other nitrogenous compounds. Typically, ammonium is assimilated into glutamine and glutamate in plant cells, which are catalyzed by glutamine synthetase (GS) and glutamine oxoglutarate aminotransferase (GOGAT), respectively [13,14]. Besides the main pathway of GS/GOGAT, plants also possess alternative routes, such as reversible amination of 2-oxoglutarate (2-OG) to produce glutamate through glutamate dehydrogenase (GDH) [15,16]. GDH can release amino nitrogen from amino acids yielding a keto acid and NH_3_ that can be separately recycled in respiration and amide formation, respectively [17]. Meanwhile GDH may catalyse in the deamination direction in tissues that converts amino acids to transport compounds at a low C:N ratio [2,17]. Although high concentrations of ammonium are toxic to plants, since it can induce proton extrusion, which is associated with ammonium uptake, changes in cytosolic pH and uncoupling of photophosphorylation in plants [14,16,18], plants have developed metabolic processes, such as nutrient remobilization, to alleviate ammonium toxicity. Toxic ammonium will be rapidly converted into organic form (amino acids) to avoid negative effects and provide nitrogenous compounds suitable for source-sink transport [5,16]. Meanwhile, pathways of second metabolites such as phenolics, flavonoids, and anthocyanins can also alleviate ammonium toxicity [14,16,19].

Therefore, based on the requirement of wastewater treatment, macrophytes that can grow and tolerate ammonium at high concentrations are preferred in CWs [4]. Recent studies showed that a C4 plant, *M. aquaticum*, can better tolerate ammonium exceeding 14 mmol/L, which indicated that this macrophyte is more adaptive to high ammonium loading wastewater, when compared with widely used macrophytes, such as *Typha latifolia*, *Juncus effusus*, and *Cyperus alternifolius* [4,20]. Plants with the C4 pathway of photosynthesis have developed a mechanism to minimize photo respiratory losses by building a higher concentration of CO_2_ around Rubisco, and they exhibit better NUE than C3 plants [21,22]. The introduction of C4-like traits into C3 plants, such as wheat and rice, therefore, has been a favored approach to improve the photosynthetic efficiency of C3 plants and enhance crop yield [21]. Additionally, studies have shown that *M. aquaticum* is easy to harvest, and its high growth rate makes it a suitable candidate used in CWs for nitrogen-rich wastewater treatment [7,9,10]. Compared with other aquatic plants such as *Canna indica*, *Hydrocotyle verticillata*, and economic crops such as corn, rice, and soybeans, *M. aquaticum* had a much higher crude protein and key amino acids content [4,13,23], which indicated that *M. aquaticum* could be used as a suitable forage for livestock. In particular, although crude protein content is lower than that in duckweed, greater content of 10 essential amino acids in *M. aquaticum* show that *M. aquaticum* could contain more high-quality protein [4]. Previous studies also showed that *M. aquaticum* could increase the activity of key enzymes involved in nitrogen metabolism such as GS, asparagine synthetase (AS), and nitrate reductase (NR) for ammonium assimilation [13,24,25]. For example, NR activity in *M. aquaticum* leaves was significantly increased to accelerate the reduction of nitrate to ammonium for ammonium assimilation when treated with low concentrations of ammonium [13]. All of these studies indicated that *M. aquaticum* possessed a highly efficient nitrogen metabolic network. However, few studies have investigated in detail the genetic regulation mechanism and key genes involved in such high NUE in *M. aquaticum*.

In this study, it is hypothesized that the high NUE by *M. aquaticum* depends on an interactive network regulating nitrogen, carbohydrate, transcription factors (TFs), and secondary metabolism. To verify this hypothesis, we grew *M. aquaticum* at five concentrations of NH_4_Cl (0, 0.1, 1, 12, and 36 mmol/L) defined as LNC, L1, L2, L3, and L4 groups for 14 days. Plant biomass, ammonium content, and different kinds of amino acids were first determined. Since plant leaves serve as nitrogen-sinks during the vegetative stage [2], a transcriptomic analysis of leaf tissues was carried out to investigate the complex molecular responses to different concentrations of ammonium. Gene Ontology (GO) enrichment and Kyoto Encyclopedia of Genes and Genomes (KEGG) pathway enrichment analysis were used to identify the important biological processes in *M. aquaticum* leaves. At last, the weighted gene correlation network analysis (WGCNA) was used to identify the most relevant gene modules related to NUE in *M. aquaticum*.

## 2. Results

### 2.1. Phenotype Characters Effected by Different Concentrations of Ammonium

As the ammonium concentration increased in the nutrient solution, the ammonium in *M. aquaticum* leaves significantly increased (*p* < 0.05) from 45 µg g^−1^ fresh weight (FW) in the LNC group to 212 µg·g^−1^ FW in the L3 group and 465 µg·g^−1^ FW in the L4 group (Table 1). Meanwhile, with increased ammonium content, the relative growth rate (RGR) significantly decreased by more than 50%, from 0.113 g·d^−1^ in the L2 group to 0.056 g·d^−1^ in the L4 group. The maximum quantum yield of photosystem II (PSII) (Fv/Fm) decreased significantly (*p* < 0.05) in the L4 group when compared to other groups. However, the length and number of fibrous roots were totally different from other phenotype characters. LNC and L1 groups own higher length and number of fibrous roots than the other groups, which indicated that *M. aquaticum* develops fibrous roots for nutrient uptake under low-ammonium conditions [4,26].

### 2.2. Effect of Ammonium on Amino Acid Content in Leaves

**Figure 1:**
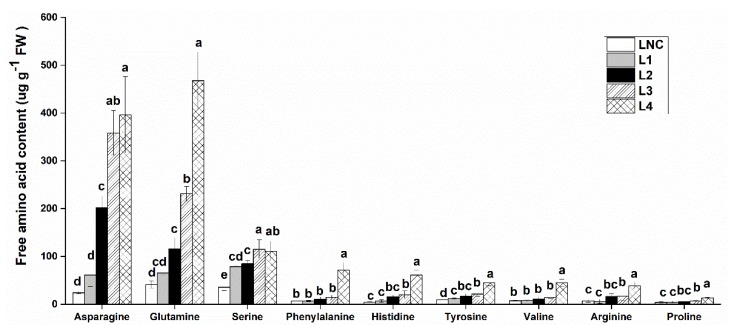
Effect of different concentrations of ammonium on the amino acid content in *Myriophyllum aquaticum* leaves. LNC: group treated with 0 mM ammonium. L1: group treated with 0.1 mM ammonium. L2: group treated with 1 mM ammonium. L3: group treated with 12 mM ammonium. L4: group treated with 36 mM ammonium values (means ± SDs) were determined from three biological replicates. Different letters above the bars indicate a significant difference at 0.05.

Changes among amino acids in response to concentrations of ammonium were quantified, and the results were shown in Figure 1. The increased ammonium in the solution also significantly induced most kinds of amino acid content in *M. aquaticum* leaves. The dominant amino acids were asparagine (Asn), glutamine (Gln), and serine (Ser), which accounted for more than 80% of the total amino acids. Asn content significantly (*p* < 0.05) increased under 1 (L2 group) and 12 mmol/L (L3 group) ammonium, and the content in the L4 group reached 16 times of the LNC group. Meanwhile, the Gln content also increased significantly, reaching 231 µg g^−1^ FW in the L3 group, and 468 µg g^−1^ FW in the L4 group, respectively. The aromatic amino acids, phenylalanine (Phe) and tyrosine (Tyr) that were substrates for phenol and flavonoid biosynthesis, were also significantly increased with greater ammonium content. Proline (Pro), which is an osmoticum and ROS scavenger often found to increase in response to abiotic stress, also showed a significant increase under high concentrations of ammonium.

### 2.3. Differential Expression of Genes to Different Concentrations of Ammonium

**Figure 2:**
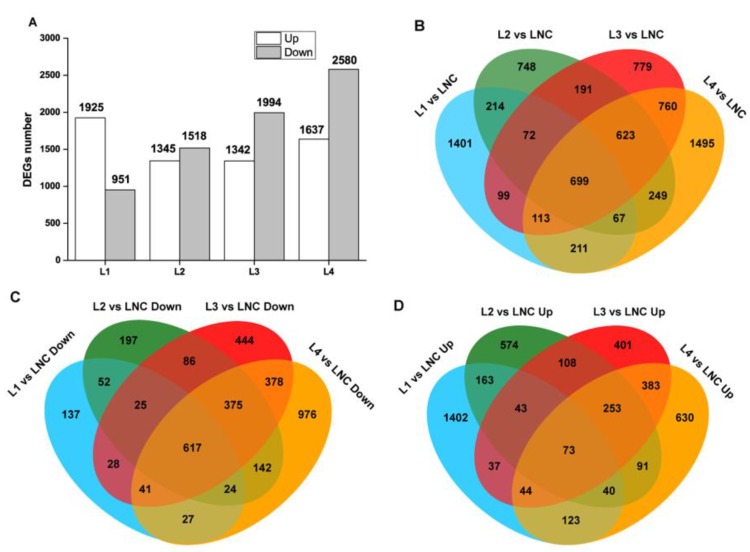
Differentially expressed genes (DEGs) among different groups in *Myriophyllum aquaticum*. (**A**) The total number of upregulated and down-regulated DEGs. (**B**–**D**) Venn diagram of all DEGs, down-regulated and up-regulated genes, respectively. LNC: group treated with 0 mM ammonium. L1: group treated with 0.1 mM ammonium. L2: group treated with 1 mM ammonium. L3: group treated with 12 mM ammonium. L4: group treated with 36 mM ammonium.

As shown in Table S1, from 15 leaves of the five groups (LNC, L1, L2, L3, and L4), a total of 76.07 million clean reads (78.34 million raw reads) were generated, pooled together, and then de novo assembled by Trinity. The transcriptome was assembled to 32,492 unigenes, with an N50 = 1617 bp. Principal component analysis (PCA) was used to capture the overall variance among the transcript data. The results illustrated that the gene expression in LNC, L1, and L2 groups were separated from each other while the L3 and L4 groups more closely resemble different PC directions with the first two PCs explaining 66% of the variation (Figure S1). The transcriptomic responses of *M. aquaticum* to different concentrations of ammonium were analyzed. The numbers of genes, whose expression was significantly (*p* < 0.05) altered based on different growth conditions, were shown in Figure 2 and Figure S2. Different concentrations of ammonium led to substantial transcriptomic responses in which 7721 genes were differentially expressed in *M. aquaticum* (Table S2). Initially, the numbers of upregulated genes were more than that of downregulated genes in the L1 group while the numbers of downregulated genes increased to 2580 in the L4 group, as the ammonium greatly increased (Figure 2A). The four groups (L1, L2, L3, and L4) shared a total number of 699 differentially expressed genes (DEGs) in common (Figure 2B), of which 617 and 73 DEGs were down-regulated (Figure 2C and Table S3) and upregulated (Figure 2D and Table S3), respectively, among the four groups when compared with the LNC group.

### 2.4. Gene Clustering Analysis

**Figure 3:**
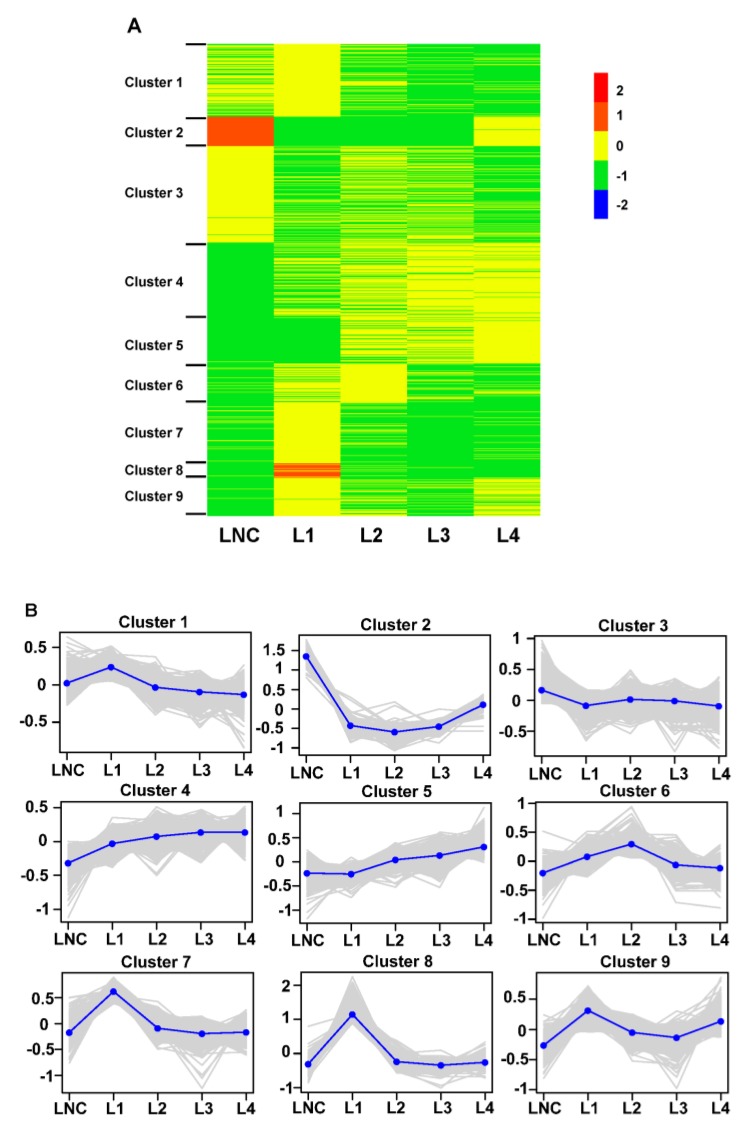
Clustering analysis of 7721 differentially expressed genes (DEGs) among different groups in *Myriophyllum aquaticum*. (**A**) Heat map of DEGs expression in different groups through K-means clustering analysis. (**B**) Expression patterns and numbers of the DEGs in the nine clusters. Color keys in (**A**) and vertical ordinate in (**B**) were presented as log10 (TPM + 1). TPM: transcripts per million reads. LNC: group treated with 0 mM ammonium. L1: group treated with 0.1 mM ammonium. L2: group treated with 1 mM ammonium. L3: group treated with 12 mM ammonium. L4: group treated with 36 mM ammonium.

The 7721 DEGs were clustered into nine co-expression modules using the *k*-means clustering method based on their expression patterns, and the results showed that the expression patterns were different among different groups (Figure 3 and Table S4). The L3 and L4 groups showed a relatively high transcript level on cluster 4 and 5, which mainly included genes encoding for proteins responsible for biosynthesis of unsaturated fatty acids (DN526726_c0_g1, DN565207_c1_g1, and DN568337_c0_g1), peroxisome (DN563687_c0_g4, DN569779_c3_g1, DN547142_c0_g1, DN570126_c7_g1) and nitrogen metabolism (DN550397_c0_g1, DN565349_c1_g2, DN566965_c2_g2, DN556336_c1_g1) while the expression of these genes was inhibited in LNC and L1 groups. The result indicated that these genes may be related to ammonium assimilation under high concentrations of ammonium. However, some genes in cluster 2 and cluster 8 were expressed at a higher level in the LNC and L1 groups compared with the other groups. For example, the expression of genes responsible for translation (DN491679_c0_g1, DN516345_c0_g2, DN523402_c0_g2, and DN558204_c4_g4), glyoxylate and dicarboxylate metabolism (DN532260_c1_g2, DN532412_c0_g1, DN543963_c1_g2, and DN554457_c1_g6) and starch and sucrose metabolism (DN554130_c1_g2, DN556040_c0_g1, DN564501_c1_g1, and DN567789_c1_g2) was highly enhanced in LNC or the L1 group. In addition, catabolic-related genes including DN516345_c0_g2, DN523744_c0_g1, DN524943_c0_g1, and DN544492_c2_g1 were also highly regulated at low concentrations of ammonium. The rest of DEGs were clustered into cluster 1, cluster 3, cluster 6, cluster 7, and cluster 9.

### 2.5. Gene Ontology and Pathway Analysis of DEGs

**Figure 4:**
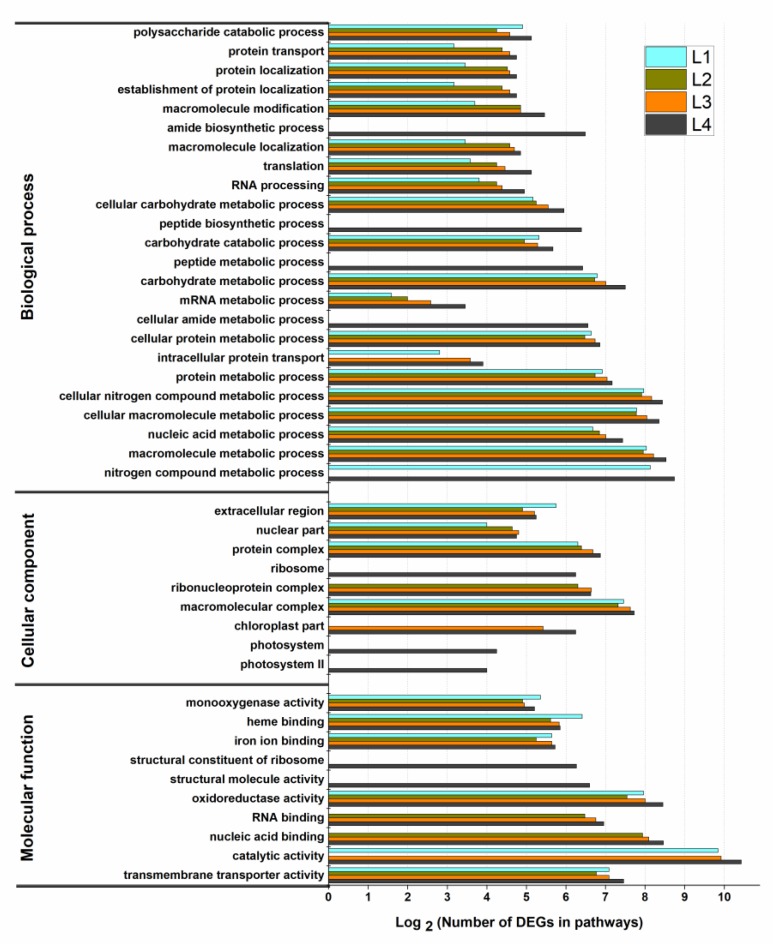
Analysis of Gene Ontology (GO) enrichment for differentially expressed genes (DEGs) among different groups in *Myriophyllum aquaticum*. LNC: group treated with 0 mM ammonium. L1: group treated with 0.1 mM ammonium. L2: group treated with 1 mM ammonium. L3: group treated with 12 mM ammonium. L4: group treated with 36 mM ammonium.

GO enrichment analysis was used for a global analysis of DEGs. The DEGs were classified into three categories of ontologies including the biological process, the cellular component, and molecular function (Figure 4). Among the biological process, the DEGs in four groups were mainly involved in the polysaccharide catabolic process (GO:0000272), protein transport (GO:0015031), protein localization (GO:0008104), establishment of protein localization (GO:0045184), macromolecule modification (GO:0043412), and amide biosynthetic process (GO:0043604). Typically, the amide biosynthetic process (GO:0043604), the peptide biosynthetic process (GO:0043043), and the peptide metabolic process (GO:0006518) were only enriched in the L4 group while the nitrogen compound metabolic process (GO:0006807) was enriched the most in L1 and L4 groups. Furthermore, the highly enriched terms included in cellular component among these groups were the extracellular region (GO:0005576), the nuclear part (GO:0044428), the protein complex (GO:0043234), and the ribosome (GO:0005840). Photosystem (GO:0009521) and photosystem II (GO:0009523) were only enriched in the L4 group while the chloroplast part (GO:0044434) was only enriched in L3 and L4 groups. Lastly, the molecular function contained 35, 18, 28, and 50 significantly (*p* < 0.05) enriched GO terms in L1, L2, L3, and L4 groups, respectively. Among these terms, monooxygenase activity (GO:0004497), heme binding (GO:0020037), and iron ion binding (GO:0005506) were highly enriched in all groups. These results indicated that a global and complex regulation network existed in *M. aquaticum*, especially in the L4 group.

**Figure 5:**
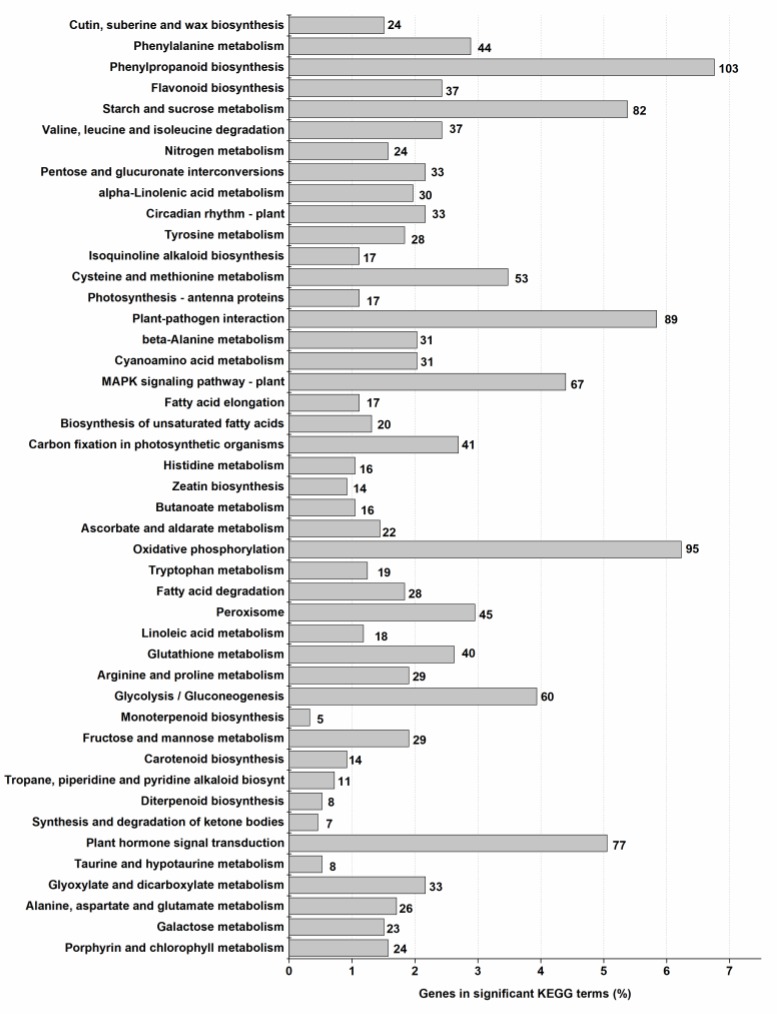
Kyoto Encyclopedia of Genes and Genomes (KEGG) analysis of 7721 differentially expressed genes (DEGs) in *Myriophyllum aquaticum* among different groups. The number of enriched genes in each term was noted.

These 7721 uni-genes were, subsequently, assigned to 126 KEGG pathways of which top 30 KEGG pathways were shown in Figure 5. Pathways of nitrogen metabolism (ko00910), starch and sucrose metabolism (ko00500), and the plant hormone signal transduction (ko04075) were significantly enriched in four groups. Genes involved in amino acid pathways in eukaryotes were also among the top 30 KEGG category, such as phenylalanine metabolism, alanine, aspartate and glutamate metabolism, carotenoid biosynthesis, valine, leucine and isoleucine degradation, tyrosine metabolism, cysteine and methionine metabolism, beta-Alanine metabolism, cyanoamino acid metabolism, histidine metabolism, tryptophan metabolism, and arginine and proline metabolism. In addition, many genes related to carbohydrate metabolisms including fructose and mannose metabolism, glycolysis/gluconeogenesis, oxidative phosphorylation, pentose and glucuronate interconversions, and galactose metabolism were also significantly (*p* < 0.05) enriched (Table S5). The pathways involved in secondary metabolites biosynthesis included phenylpropanoid biosynthesis, stilbenoid, diarylheptanoid and gingerol biosynthesis, flavonoid biosynthesis, diterpenoid biosynthesis, anthocyanin biosynthesis, and isoquinoline alkaloid biosynthesis were also significantly (*p* < 0.05) enriched under the KEGG category. These results indicated that multiple pathways may collectively contribute to ammonium assimilation within a complex and interactive network.

### 2.6. Differential Expression Behavior of Genes Involved in Nitrogen and Carbohydrate Metabolisms

A total of 32 genes involved in nitrogen metabolism related to nitrogen transportation, assimilation, and remobilization were identified. The expression analysis revealed that most of these genes were differentially expressed in the groups treated with ammonium (Table S2). At low concentrations of ammonium, genes encoding for ammonium transporters (AMT) such as AMT1.2 (DN547206_c0_g1) and AMT3.1 (DN557461_c1_g1) were upregulated. However, when ammonium concentration increased, genes encoding enzymes involved in ammonium transportation were downregulated. Even though little nitrate existed in the culture solution, nitrate transporters such as NRT2.4 (DN565032_c0_g2) and NRT2.5 (DN565032_c0_g1) were also expressed as a similar expression pattern as AMTs. Meanwhile, various isoforms of genes encoding enzymes of nitrogen assimilation, including GS (DN543963_c1_g2, DN564519_c0_g1), GOGAT (DN565349_c1_g2, DN565349_c1_g3), GDH (DN563092_c2_g3) and AS (DN561591_c5_g1, DN549208_c0_g1), NR (DN566965_c2_g2), and nitrite reductase (NiR, DN570758_c9_g3) were detected in all treated groups. Most of the genes related to nitrogen assimilation showed no significant variations in the L1 group compared with the LNC group. However, the expressions of these genes were significantly (*p* < 0.05) upregulated at high concentrations of ammonium (L3 and L4 groups).

Genes responsible for carbohydrate metabolism, including fructose-bisphosphate aldolase (DN557851_c2_g2, DN558627_c1_g1), phosphofructokinase (DN547826_c1_g1, DN568973_c1_g2), 6-phosphogluconolactonase (DN566576_c1_g4), transketolase (DN537175_c0_g2), and phosphoenolpyruvate carboxylase (DN546635_c0_g1, DN566112_c2_g2) were found to be differently regulated under different concentrations of ammonium (Table S2). Genes encoding for phosphofructokinase and fructose-bisphosphate aldolase were upregulated and downregulated in all treated groups, respectively, while encoding genes for 6-phosphogluconolactonase and phosphoenolpyruvate carboxylase were upregulated and then downregulated when the ammonium concentration increased.

### 2.7. Identification of Differentially Expressed TFs

**Figure 6:**
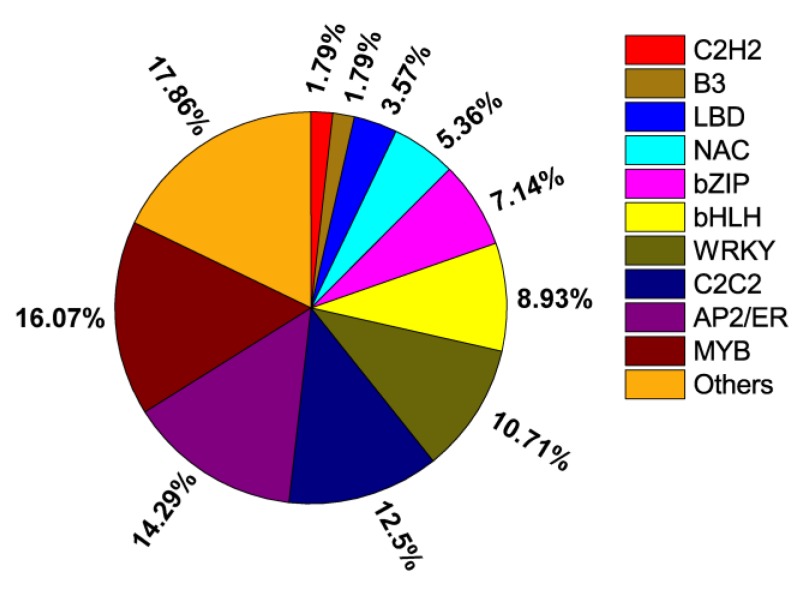
Differentially expressed transcription factors in the four groups. Ten of the most enriched transcription factor families were shown.

TFs play important roles in plant signalling pathways. A total number of 343 genes encoding TFs across four groups were identified from DEGs (Table S6). These TFs could be classified into 25 families, mainly including MYB (16.07%), AP2/ERF (14.29%), C2C2 (12.5%), WRKY (10.71%), bHLH (8.93%), bZIP (7.14%), NAC (5.36%), LBD (3.57%), C2H2 (1.79%), and B3 (1.79%) families (Figure 6). Even though some uni-genes were annotated to the same TF family, such as the predicted MYB encoded genes DN541813_c0_g1 and DN569529_c3_g1, their expression patterns were different among different groups when the ammonium concentration increased (Table S2). These results indicated an extremely intricate and complex transcriptional network present for ammonium assimilation at different concentrations of ammonium.

### 2.8. Weighted Gene Co-Expression Network Analysis

**Figure 7:**
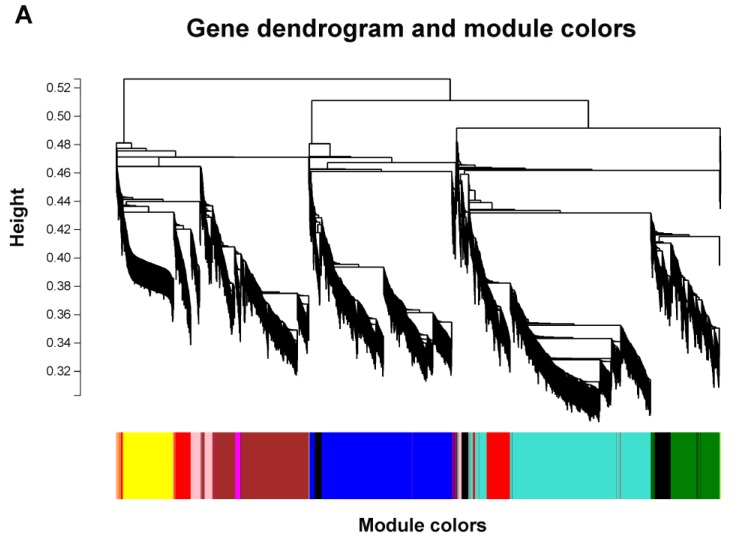

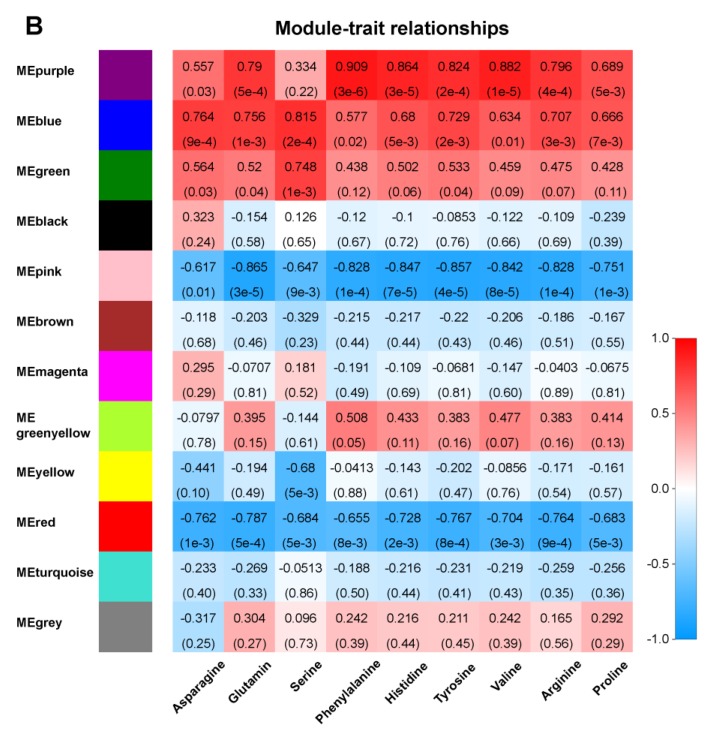
Weighted correlation network analysis of differentially expressed genes (DEGs) in *Myriophyllum aquaticum*. (**A**) Hierarchical clustering tree showing co-expression modules. Each leaf in the tree represents one gene. The major tree branches constitute 10 modules labelled by different colors. (**B**) Module-trait relationship. The left lane indicates 10 module Eigen genes. The right lane indicates the module-trait correlation from −1 to 1.

To analyze the genes correlated with ammonium assimilation at a network-level, WGCNA was performed to construct a weighted correlation network with the differentially expressed genes [27]. In this study, 10 modules were identified from the RNA-sequence data (Figure 7A), and module eigen genes for these modules were linked to different amino acids. The module-trait relationship showed that the modules MEblue and MEpurple (positive correlation modules) were highly positively correlated with amino acid content except for Ser (Figure 7B), while the modules MEred and MEpink (negative correlation modules) were highly negatively correlated, which were significantly correlated (*p* ≤ 0.05) with all kinds of amino acids. The modules MEblue and MEpurple contained 1522 and 48 genes, respectively. Meanwhile, 529 and 261 genes were identified in the modules MEred and MEpink (Table S7). Correlation analysis between different modules indicated that correlation coefficients between MEblue and MEpurple or between MEred and MEpink were relatively higher than correlation coefficients between positive correlation modules and negative correlation modules (Figure S3). The highest correlation coefficients existed between MEgrey and MEgreenyellow as well as between MEmagenta and MEbrown modules, which reached 0.873 and 0.853, respectively.

To further understand the mechanism of ammonium assimilation in *M. aquaticum*, the GO enrichment and KEGG pathway of uni-genes in the positive and negative correlation modules were independently analyzed. The uni-genes in positive correlation modules were mainly enriched in oxoacid metabolic process (GO:0043436), organic acid metabolic process (GO:0006082), translation (GO:0006412), and the cellular protein metabolic process (GO:0044267) (Table S8) while the highly enriched terms of negative correlation modules were associated with photosynthesis, light harvesting (GO:0009765), protein-chromophore linkage (GO:0018298), protein refolding (GO:0042026), and the microtubule-based process (GO:0007017). The top 30 enriched KEGG pathways of positive and negative correlation modules were illustrated in Figure S4. Most of the pathways in the positive correlation modules were significantly (*p* < 0.05) enriched while only one pathway (photosynthesis-antenna proteins) were significantly (*p* < 0.05) enriched in the negative correlation modules. The most significantly (*p* < 0.05) enriched pathways in KEGG analysis of the positive and negative modules were valine, leucine, and isoleucine degradation and photosynthesis-antenna proteins, respectively.

### 2.9. Validation of Differentially Expressed Genes

To verify the RNA-seq data, qRT-PCR was conducted on nine selected genes involved in different metabolisms. The primers used for qRT-PCR were listed in Table S9. The qRT-PCR results indicated that the relative expression levels of nine selected genes were largely consistent with the transcriptome profile, which is shown by the RNA-seq data (Figure S5).

## 3. Discussion

Excessive input and accumulation of nitrogen waste, especially ammonium, has considerably deteriorated the water quality of many aquatic ecosystems. Except traditional techniques deployed in wastewater treatment plants, many advanced treatment approaches were broadly developed and studied. CWs, due to their environmentally-friendly properties, have been universally used for wastewater advanced treatment. In CWs, the macrophytes are one of the key factors affecting the treatment effects. Therefore, it is preferential if certain macrophytes can withstand and efficiently treat wastewater of high ammonium concentration [4]. Recent studies showed that *M. aquaticum* was one of the macrophytes that could not only tolerate wastewater of high ammonium concentration, but also efficiently transfer external ammonium into plant tissues and transform into constitutive amino acids [4,13,23]. Such high NUE has caught our attention, and the genetic mechanism underlying the high NUE was investigated through transcriptome and weighted gene co-expression network analysis in this study.

### 3.1. Nitrogen Metabolism and Amino Acid Synthesis

It is believed that the high NUE in *M. aquaticum* is closely linked to highly efficient nitrogen metabolic systems, including ammonium transport and assimilation systems. Although studies had investigated the function of some key enzymes in ammonium assimilation of *M. aquaticum* [13], a global investigation on the gene regulation mechanism has not been carried out. In this study, the differential expression of genes closely related to ammonium transportation and assimilation at different concentrations of ammonium indicated that these functional genes would be one of the crucial factors contributing to differential responses to ammonium treatment. Excessive ammonium is toxic to *M. aquaticum*. Therefore, the ammonium transportation and assimilation systems should be synergistically regulated [1]. In *Arabidopsis*, three dominant AMTs (AMT1;1, AMT1;2, and AMT1;3) import 95% of the ammonium at low ammonium concentrations and AMT1;3 is critical for ammonium-triggered lateral root branching [1,12,28]. In *M. aquaticum*, genes (DN547206_c0_g1 and DN557461_c1_g1) encoding AMT1;2 and AMT3;1 were upregulated at low concentrations of ammonium and downregulated at high concentrations of ammonium. Such responsive regulations on gene expression may help *M. aquaticum* to better cope with different surrounding ammonium concentrations, which is similar to the gene regulation in *Arabidopsis* [13,20]. Genes encoding NRTs (DN565032_c0_g1 and DN565032_c0_g2) were downregulated at high concentrations of ammonium and upregulated at low concentrations of ammonium in *M. aquaticum*. Previous studies showed that NRTs played an important role in unfavorable environment adaptation of plants [29,30]. Meanwhile, deletion of *NRT1;1* could significantly increase the ammonium tolerance in *Arabidopsis* [30]. In this study, downregulation of genes encoding NRTs at high concentrations of ammonium might also assist *M. aquaticum* to resist ammonium toxicity while upregulation of genes encoding NRTs at low concentrations of ammonium might produce more NRTs for nitrate uptake to alleviate nitrogen starvation.

Ammonium transportation into *M. aquaticum* tissues is much more likely to induce responses for ammonium assimilation into amino acids. Previous studies reported the increase in enzyme activity for ammonium assimilation, including GOGAT, AS, and GS [1,13]. In this study, several uni-genes encoding GDH and GOGAT were induced at high concentrations of ammonium (Table S2). GDH is a catabolic enzyme mainly contributing to amino acid breakdown under C deficient conditions to provide 2-OG for the citric acid (TCA) cycle [17] and to ammonium assimilation [14,15]. Even though it was reported that the primary role of GDH under non-stress conditions was catalyzing glutamate decomposition [31,32], several studies demonstrated that GDH played an important assimilating role under stress conditions of high concentrations of ammonium [16,18,33]. Additionally, high concentrations of ammonium also substantially stimulated the expression of AS (DN549208_c0_g1), which indicates its close association with ammonium assimilation in *M. aquaticum*, as reported in a previous study [13,24]. Although GS was one of the main contributors to primary nitrogen assimilation and protein storage in plant tissues [16,17,34], the expression of GS was dramatically downregulated at high concentrations of ammonium. Considering the significantly increased Gln content at high concentrations of ammonium, one explanation was that a post-transcriptional regulation of GS activity existed in *M. aquaticum* [12,17,34]. Another explanation was that GS activity could be upregulated and then downregulated during the long period (14 days) cultivation with high concentrations of ammonium, which needed to be further confirmed. Besides the increase in the nitrogen scavenging mechanism, upregulation of the amidase encoding gene (DN569606_c1_g2) indicated that *M. aquaticum* could degrade organic nitrogen sources such as amide from intracellular or possible extracellular sources to produce ammonium when facing nitrogen deprivation [15].

Amino acids are the main long-distance nitrogen transport forms in plant tissues and they also regulate inorganic nitrogen transportation and assimilation [1,2]. Comparative transcriptome analysis suggested that increased expression of nitrogen metabolism genes mainly occurred at high concentrations of ammonium. Aspartate aminotransferase (AspAT) responsible for reversible transfer of the amino group of aspartate to a-ketoglutarate [35] also played important roles in providing precursors for biosynthesis of major nitrogen transport molecules such as asparagine and ureides, and of amino acids of the aspartate family [21,35]. In this study, the gene (DN553386_c0_g1 and DN558157_c0_g1) encoding AspAT was significantly upregulated at high concentrations of ammonium, which indicated an enhanced process for amino acids biosynthesis. Alanine aminotransferase (ALT, DN537652_c1_g1) was also upregulated at high concentrations of ammonium. ALT is an assimilatory enzyme for the synthesis of alanine and 2-OG from pyruvate and glutamate vice-versa [21], which is important for maintaining the carbohydrate and nitrogen metabolism in the plant system [36]. Overexpression of ALT in some crops, including rice and barley, could increase plant biomass, seed yield, and NUE [34,37]. The induction of the ALT gene in *M. aquaticum* in response to high concentrations of ammonium is likely a strategy to adjust the nitrogen and carbon shuttle. Moreover, some other genes involved in amino acid biosynthesis were also upregulated at high concentrations of ammonium such as phosphoserine phosphatase (DN542100_c1_g1) and tryptophan synthase (DN539934_c0_g2).

In general, the responsive regulation of nitrogen-assimilatory genes in *M. aquaticum* at high concentrations of ammonium was one of the critical factors contributing to its high tolerance to ammonium and high NUE.

### 3.2. Carbohydrate and Energy Pathways

The expression of the C4-specific pepcase can promote photosynthesis [21]. However, genes encoding pepcase (DN568937_c0_g1 and DN568937_c0_g2) in this study were significantly repressed at high concentrations of ammonium. Additionally, some genes encoding the light-harvesting complex (LHC) (DN561030_c5_g4) were also significantly downregulated (*p* < 0.05) at high concentrations of ammonium, which were reported when facing other abiotic stresses [15,38]. Previous studies also showed that ammonium could trigger leaf chlorosis while the AMOS1/EGY1 nitrogen-dependent response recruits the ABA signaling pathway to prevent leaves from chloroplast damage [12,26]. The gene (DN553399_c0_g1) encoding alpha-amylase, which is one of the important enzymes for starch remobilization under stress conditions [39], was significantly upregulated (*p* < 0.05) at high concentrations of ammonium. Thus, degradation of starch might be accelerated to release more glucose and acetyl-CoA to facilitate carbohydrate metabolism and ammonium assimilation in *M. aquaticum*, as it was reported in other plant models [15,16,28,39,40]. 

Furthermore, carbohydrate metabolism is also important for ammonium assimilation by providing organic acids and energy, which is indispensable in amino acid synthesis [16,28,41]. Genes encoding citrate synthase (DN544376_c2_g4 and DN570545_c7_g1) and isocitrate dehydrogenase (DN553834_c2_g1) was significantly upregulated (*p* < 0.05) at high concentrations of ammonium. These enzyme functions are the key processes of the TCA cycle [28,32], which regulates the energy and NAD(P)H in plant tissues and also supplies substrates for amino acid synthesis [41]. It was well studied that, when responding to high concentrations of the ammonium supply, a series of reactions, including ammonium-dependent enhancement of carbon input and respiration, would systematically occur to synthesize amino acids [11,28,41]. Thus, it was postulated that increased degradation of starch, along with the induction of the TCA cycle, could provide ammonium stressed cells with enough energy as well as many other substrates for amino acid synthesis in *M. aquaticum*.

### 3.3. Involvement of TF Families and Protein Kinases in the Ammonium Mediated Signalling Response

A number of TFs and protein kinases, including MYB, WRKY, NAC, bHLH, bZIP, and AP2, were involved in nitrogen mediated signalling and regulatory networks [12,22,42,43] including some that were even involved in long-distant signalling between the leaf and the root [42]. In this study, comparative analysis on transcription factors that were significantly regulated responding to different concentrations of ammonium demonstrated that MYB, AP2, and WRKY TFs were the most abundant TF families (Figure 6). Previous studies proved the involvement of MYB TFs in the expression of nitrogen transporters and assimilatory genes [43], as well as their important roles in GS isogenes regulation in *Arabidopsis* and rice [34]. Although the exact role of AP2 in nitrogen-related signaling mediation is not known, involvement of AP2 adapting to nitrogen deficiency was reported in a previous study [37]. Dof1 TFs can increase nitrogen assimilation in many plants such as maize and tobacco [37]. Two Dof1 genes in *M. aquaticum* were identified including one that (DN552745_c4_g1) was significantly upregulated (*p* < 0.05) at low ammonium concentrations (L1 and L2 group). The other (DN545277_c1_g1) was upregulated at high ammonium concentrations (L4 group), which indicated a universal stimulation of nitrogen assimilation in *M. aquaticum*. In general, the differential expression of different transcription factors in response to different concentrations of ammonium may also be one of the factors responsible for high NUE in *M. aquaticum*.

Protein kinases also played important roles in nitrogen signalling [31,44,45]. In our transcriptome data, histidine kinase 1 (DN569908_c5_g2, DN569908_c5_g1 and DN569944_c1_g1) was upregulated at a low ammonium concentration. Histidine kinases took part in a variety of plant signalling responses to abiotic stress [22,46]. Therefore, upregulation of histidine kinase 1 in response to low ammonium treatment may also reveal its involvement in ammonium-mediated signalling in *M. aquaticum*. In plants, regulation of nitrogen sensing and transportation by calcium signalling requires Ca^2+^ dependent protein kinases (CDPKs) and CBL-interacting protein kinases (CBL/CIPKs) [1,31,44,45]. In this study, eight CDPKs (CDPK 3, 5, 8, 11, 16, 28, 29, and 32) and four CIPKs (CIPK 1, 5, 11, and 12) were differentially regulated in response to ammonium treatment. The CDPK11, CDPK16, and CDPK28 were commonly induced at low concentrations of ammonium, whereas CDPK8 and CDPK32 were specifically induced at high concentrations of ammonium. In *Arabidopsis*, CDPK was involved in the activation of anion channels such as SLAC1 [47]. Therefore, the induction of CDPK in *M. aquaticum* responding to ammonium treatment indicated its role in nitrogen sensing and transportation. As for CIPKs, CIPK 11 and CIPK 12 were significantly induced (*p* < 0.05) at low concentrations of ammonium while CIPK 1 and CIPK 11 were significantly induced (*p* < 0.05) at high concentrations of ammonium. However, CIPK 5 was significantly repressed at high concentrations of ammonium, which indicated a strategy to ammonium stress [44,45]. Previous studies emphasized the involvement of CIPKs in regulating the nitrogen-mediated response in plants [31,44]. Therefore, it could be hypothesized that CIPKs identified in this study might be important components of nitrogen signalling in *M. aquaticum*.

### 3.4. Regulation of Secondary Metabolite Pathways at Different Ammonium Conditions

Phenols serve a wide range of functions as ROS scavengers, lipid peroxidation inhibitors, or electron donors [14,19,48]. However, whether phenols, especially phenolics, flavonoids, and anthocyanins, play any roles in ammonium assimilation for macrophytes, particularly *M. aquaticum*, remains unclear. KEGG analysis indicated that genes for phenylpropanoid and flavonoid biosynthesis were significantly enriched (*p* < 0.05) in the 7721 DEGs, which ranked the third and fourth most enriched, respectively. In this study, most genes involved in phenylpropanoid biosynthesis, including cinnamoyl-CoA reductase (DN563398_c1_g1 and DN562056_c0_g1) and cinnamyl-alcohol dehydrogenase (DN562714_c1_g1 and DN559807_c1_g3), were significantly upregulated (*p* < 0.05) at high concentrations of ammonium. This was also reported in previous studies [18,19]. Accelerated synthesis of salicylic acid through the phenylpropanoid pathway that utilized chorismate, which is the product of the shikimate pathway, as a precursor, indicated a hormone signaling regulation at high concentrations of ammonium [22]. The end products of phenylpropanoid biosynthesis are different kinds of lignin, which is the constitutive components of the cell wall, plant mechanical support, and the defense system [18]. Additionally, the stimulation of the phenylpropanoid pathway was considered to be a common response to some abiotic stress such as drought, salinity, ozone intoxication, and heavy metals [14,16,18]. Although lignin does not contain nitrogen, the first step in the phenylpropanoid pathway, driven by phenylalanine ammonia lyase (PAL), requires Phe whose synthesis was significantly enhanced (*p* < 0.05) at high concentrations of ammonium (Figure 1). PAL genes (DN559452_c0_g1) were more expressed at high concentrations of ammonium in this study while ammonium released from Phe by PAL could be further assimilated by GS [16]. These results indicated a close correlation between nitrogen assimilation and phenylpropanoid biosynthesis. Under nitrogen deficiency conditions, the phenylpropanoid production was adjusted to the anthocyanin pathway, which could help plants adapt to nitrogen deficiency conditions [16,19,48]. In this study, genes for anthocyanins biosynthesis, such as anthocyanidin synthase (DN555985_c2_g1), anthocyanidin reductase (DN559950_c0_g4), and leucoanthocyanidin reductase (DN555793_c0_g1), were significantly upregulated (*p* < 0.05) at low concentrations of ammonium, and the correspondingly enriched anthocyanin pathway might be a strategy for *M. aquaticum* to adapt ammonium limiting conditions [48]. The enhanced synthesis of phenylpropanoids and their derivatives such as phenolics, anthocyanins, and lignin at different ammonium concentrations further emphasized the importance of these secondary metabolites for *M. aquaticum* adapting to various ammonium concentrations.

## 4. Materials and Methods

### 4.1. Experimental Design

This study was conducted in a greenhouse under a 12/12 h dark/light photo-period at a stable temperature (25–30 °C) at the Research Center for Eco-Environmental Sciences, Chinese Academy of Sciences, Beijing. *M. aquaticum* seedlings with a uniform length of 50 ± 3 cm were cultivated in the containers (0.5 m × 0.4 m × 0.4 m) that consisted of 25 L 50% modified Hoagland solution. Nutrient solution formulation of 50% modified Hoagland solution was shown in Table S10. *M. aquaticum* treated with 0.1, 1, 12, and 36 mmol/L NH_4_Cl were defined as L1, L2, L3, and L4 groups, respectively, while a negative control (LNC) was treated with 0 mmol/L NH_4_Cl. The pH of the culture solution was adjusted to 6.0 ± 0.1 with 1 mol/L KOH. The culture solution was exchanged every 2 days to maintain the pH and nutrient concentrations. After 14 days of cultivation, plants in each container were harvested. Leaf samples were rinsed with distilled water three times and ground with liquid nitrogen, as previously described [5,19]. The ground samples were fully mixed, part of which was used for amino acids determination, while the rest was used for RNA extraction.

### 4.2. Phenotype Characters Analysis

The ammonium content in plant tissues was measured using the method described previously [13] and expressed as µg g^−1^ FW. To determine plant growth, plants were harvested on day 14 and then measured for RGR, as previously described [20]. Fv/Fm was measured using an LI-6400 portable photosynthesis system (LI-COR Inc., Lincoln, Nebraska, NE, USA) [49]. An L-8900 high-speed amino acid analyser (Hitachi, Japan) determined the amounts of free amino acids [19]. 

### 4.3. RNA Extraction, Sequencing, and Annotation

Leaf samples were fully ground with liquid nitrogen, and 200 mg sample was used for total RNA extraction with an EASYspin Plus CompleSx Plant RNA Kit following the manufacturer’s instruction (Aidlab Biotech, Beijing, China). Then the integrity and purity of the total RNA quality was determined by a 2100 Bioanalyser (Agilent Technologies, Palo Alto, CA, USA) and quantified using the NanoDrop-2000 (Thermo Scientific, Wilmington, DE, USA). Only a high-quality RNA sample (OD260/280 = 1.8~2.2, OD260/230 ≥ 2.0, RIN ≥ 8.5, 28S:18S ≥ 1.0, >2 μg) was used to construct the sequencing library. The NEBNext^®^ Ultra™ RNA Library Prep Kit for Illumina^®^ (#E7530L, NEB, Ipswich, MA, USA) was used for the sequencing library construction [46]. Three biological replicates of each group were used for RNA-seq analysis. Poly(A) mRNA was purified from total RNA using oligo-dT-attached magnetic beads and then fragmented by using the fragmentation buffer. Taking these short fragments as templates, double-stranded cDNA was synthesized by using a SuperScript double-stranded cDNA synthesis kit with random hexamer primers. Then the synthesized cDNA was subjected to end-repair, phosphorylation, and “A” base addition, according to NEB’s library construction protocol. Libraries were size selected for cDNA target fragments of 200–300 bp on 2% Low Range Ultra Agarose followed by PCR amplified using Phusion DNA polymerase (NEB, USA) for 15 PCR cycles.

After the index-coded samples were clustered, the libraries were sequenced on a HiSeq X Ten platform, and 150 bp paired-end reads were generated. The raw RNA-seq reads were deposited in the National Center for Biotechnology Information (NCBI) Sequence Read Archive (SRA, study accession number: SRR8399985-SRR8399999). The raw paired end reads were trimmed and quality controlled by SeqPrep (https://github.com/jstjohn/SeqPrep) and Sickle (https://github.com/najoshi/sickle) with default parameters. Then clean data from the samples were used to complete *de novo* assembly with Trinity (http://trinityrnaseq.sourceforge.net/) [50]. All the assembled transcripts were searched against the NCBI protein non-redundant, String, and KEGG databases using BLASTX to identify the proteins that had the highest sequence similarity with the given transcripts to retrieve their function annotations and a typical cut-off E-values less than 1.0×10^−5^ was set. The BLAST2GO (http://www.blast2go.com/b2ghome) [51] program was used to get GO annotations of unique assembled transcripts for describing biological processes, molecular functions, and cellular components. Metabolic pathway analysis was performed by using the KEGG database (http://www.genome.jp/kegg/) [52].

### 4.4. Gene Differential Expression and Enrichment Analysis

HTSeq v0.6.0 was used to count reads for each gene in each sample, and transcripts per million reads (TPM) were calculated to estimate the expression level of genes [53]. Uni-genes with a total TPM across the five groups of less than five or uni-genes with more than three groups having no detectable expression were filtered prior to clustering [53]. An estimated false discovery rate (FDR) was assigned to each gene and adjusted with the Benjamini and Hochberg approach to control the FDR [54]. Genes with an FDR ≤ 0.05 and a |log2 ratio| ≥ 1 were identified as DEGs.

### 4.5. Weighted Gene Correlation Network Analysis of Nitrogen Responding Genes

All the differentially expressed genes were assigned to build a correlation network using the WGCNA R package (https://horvath.genetics.ucla.edu/html/CoexpressionNetwork/Rpackages/WGCNA/). The adjacency matrix was generated by calculating the Pearson’s correlations among DEGs. The power β was determined based on the scale-free topology criterion [27]. The modules were detected as branches of the dendrogram and a cut-off height of 0.25 was used to merge the branches of modules. The module Eigen gene (ME) value was calculated and the association of modules with amino acid content was estimated.

### 4.6. Gene Expression Validation

To further evaluate the reliability of the RNA-seq results, nine genes involved in nitrogen, carbohydrate, TFs, and secondary metabolisms were selected for qRT-PCR to assess their expression in response to different concentrations of ammonium. Total RNA was extracted from three replicates for each treatment and used for qRT-PCR. The β-actin encoding gene was used as an internal control. The relative gene expression level was calculated as described previously [55].

### 4.7. Statistical Analysis

IBM SPSS 20.0 software (IBM Corp., New York, NY, USA) was used for data analysis. One-way analysis of variance (ANOVA) with the Tukey’s test was applied to identify significant differences between groups and the significant level was 0.05.

## 5. Conclusions

In this study, a global transcriptomic analysis on *M. aquaticum* in response to a serial of ammonium treatments was performed to investigate the mechanism behind high NUE and ammonium assimilation by *M. aquaticum*. Ammonium was efficiently assimilated into key amino acids in different treatment groups, especially in groups of high concentrations of ammonium. A number of genes involved in nitrogen-transport, assimilation, and remobilization in response to various ammonium conditions made the dominant contributions to high NUE in these groups. Meanwhile, genes involved in the carbohydrate, TFs, and secondary metabolism also played important roles in regulating for high NUE. In addition, the co-expression network analysis of DEGs identified several ammonium regulatory modules that might be associated with phenylpropanoid and amino acid metabolism in *M. aquaticum*. Future work will focus on functionally characterizing the ammonium responsive transcripts, especially from high NUE groups, which may be the potential candidates to improve NUE of *M. aquaticum* and related wetland plant species.

## Figures and Tables

**Table 1:** Phenotype character analysis of *Myriophyllum aquaticum* under different concentrations of ammonium. RGR: relative growth rate. Fv/Fm: maximum quantum yield of PSII. FW: fresh weight. DW: dry weight. LNC: group treated with 0 mM ammonium. L1: group treated with 0.1 mM ammonium. L2: group treated with 1 mM ammonium. L3: group treated with 12 mM ammonium. L4: group treated with 36 mM ammonium. The values are the means ± SDs (*n* = 3). Different letters indicate a significant difference at 0.05.

Groups	Ammonium Concentration (ug g^−1^ FW)	RGR (g d^−1^ FW)	Fv/Fm	Root Length (cm)	Root Number
LNC	44.752 ± 8.037 ^d^	0.044 ± 0.008 ^c^	0.805 ± 0.006 ^b^	17.12 ± 1.343 ^a^	29 ± 2 ^a^
L1	57.655 ± 7.134 ^d^	0.08 ± 0.017 ^abc^	0.818 ± 0.006 ^ab^	18.243 ± 1.136 ^a^	30 ± 3 ^a^
L2	100.94 ± 8.316 ^c^	0.113 ± 0.034 ^a^	0.833 ± 0.004 ^a^	12.183 ± 0.949 ^b^	20 ± 3 ^b^
L3	212.258 ± 13.871 ^b^	0.088 ± 0.004 ^ab^	0.832 ± 0.004 ^a^	7.397 ± 0.728 ^c^	8 ± 4 ^c^
L4	464.647 ± 44.995 ^a^	0.056 ± 0.031 ^bc^	0.779 ± 0.018 ^c^	5.693 ± 1.696 ^c^	4 ± 3 ^c^

## Supplementary Materials

Supplementary materials can be found at https://www.mdpi.com/1422-0067/20/7/1587/s1. Figure S1: Principal component analysis of the gene expression of *Myriophyllum aquaticum* at different concentrations of ammonium. Figure S2: Volcano plots of the differentially expressed genes (DEGs) between the control and treated *Myriophyllum aquaticum*. Figure S3: Heatmap of correlation analysis among 10 modules. Figure S4: Scatterplot of top 30 Kyoto Encyclopedia of Genes and Genomes (KEGG) pathway enrichment for differentially expressed genes (DEGs) in the positive and negative modules. Figure S5: qRT-PCR validation of differentially expressed genes in *Myriophyllum aquaticum* at different concentrations of ammonium. Table S1: RNA-seq data analysis of *Myriophyllum aquaticum*. Table S2: Change of expression and annotation of all the DEGs in the treated groups of *Myriophyllum aquaticum*. Table S3: Downregulated or upregulated differentially expressed genes (DEGs) of *Myriophyllum aquaticum* in all four treated groups. Table S4: Genes expression and annotation in the nine clusters of *Myriophyllum aquaticum*. Table S5: Kyoto Encyclopedia of Genes and Genomes (KEGG) analysis of DEGs enriched in ammonium treated groups. Table S6: Differentially regulated transcription factors of *Myriophyllum aquaticum*. Table S7: Differentially expressed genes (DEGs) of *Myriophyllum aquaticum* enriched in different modules. Table S8: Gene Ontology (GO) analysis of differentially expressed genes (DEGs) enriched in the positive and negative modules. Table S9: Primers used for qRT-PCR in *Myriophyllum aquaticum* at different concentrations of ammonium. Table S10: Nutrient solution formulation of 50% modified Hoagland solution.
