# Supplementary material for: Transcriptomic Sequencing and Co-Expression Network Analysis on Key Genes and Pathways Regulating Nitrogen Use Efficiency in Myriophyllum aquaticum
Source: Int J Mol Sci. 2019 Mar 29;20(7):1587. doi: 10.3390/ijms20071587 (PMC6480359; doi:10.3390/ijms20071587)
Supplement: Supplementary file 1 [file ijms-20-01587-s001.zip › Supplementary Materials/Supplementary Figures.docx]

**Key genes and pathways related to nitrogen use efficiency in *Myriophyllum aquaticum* revealed by RNA sequencing and co-expression network analysis**

**Rui Wang^1,2^, Shengjun Xu^1,2^, Cancan Jiang^1,2^, Haishu Sun^1,2^, Shugeng Feng^1,2^, Sining Zhou^1,2^, Guoqiang Zhuang^1,2^, Zhihui Bai^1,2^ and Xuliang Zhuang^1,2,^***

^1^ Key Laboratory of Environmental Biotechnology, Research Center for Eco-Environmental Sciences, Chinese Academy of Sciences, Beijing 100085, China;

^2^ College of Resources and Environment, University of Chinese Academy of Sciences, Beijing 100049, China;

* Correspondence: xlzhuang@rcees.ac.cn; Tel.: +86-10-6284-9193

The following Supplementary Figures is available for this article:

**
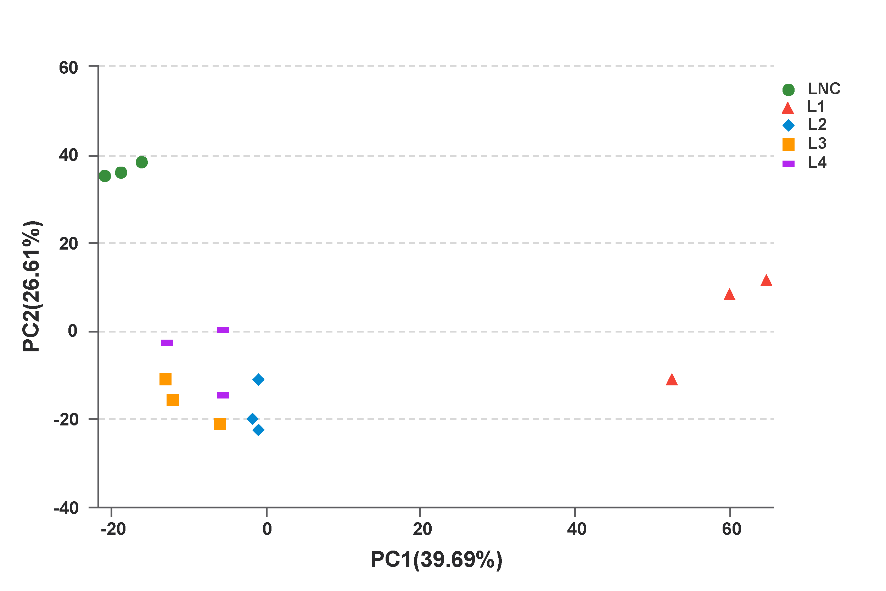
**

**Figure S1.** Principal component analysis of the gene expression of *Myriophyllum* *aquaticum* at different concentrations of ammonium. The first two PCs explain 66% of the overall variation, with PC1 primarily accounting for differences between L2 and L3 groups. PC2 primarily separates samples between LNC and L1 groups. LNC: group treated with 0 mM ammonium; L1: group treated with 0.1 mM ammonium; L2: group treated with 1 mM ammonium; L3: group treated with 12 mM ammonium; L4: group treated with 36 mM ammonium.

**
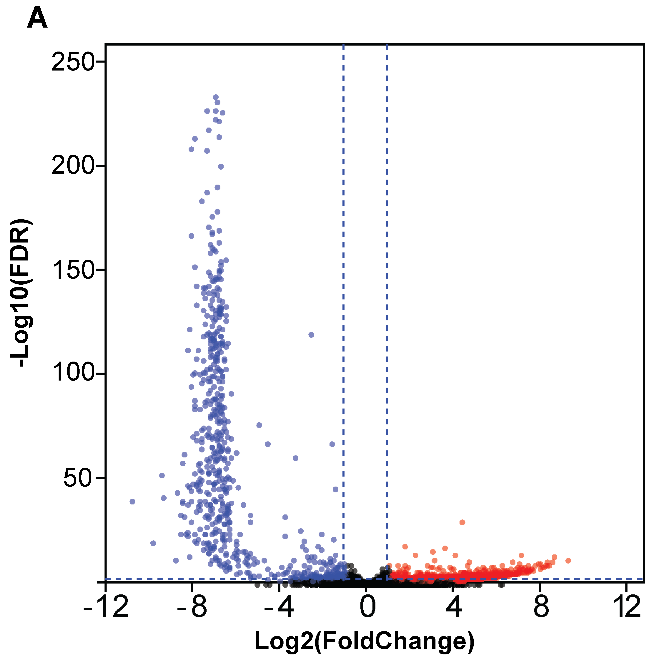

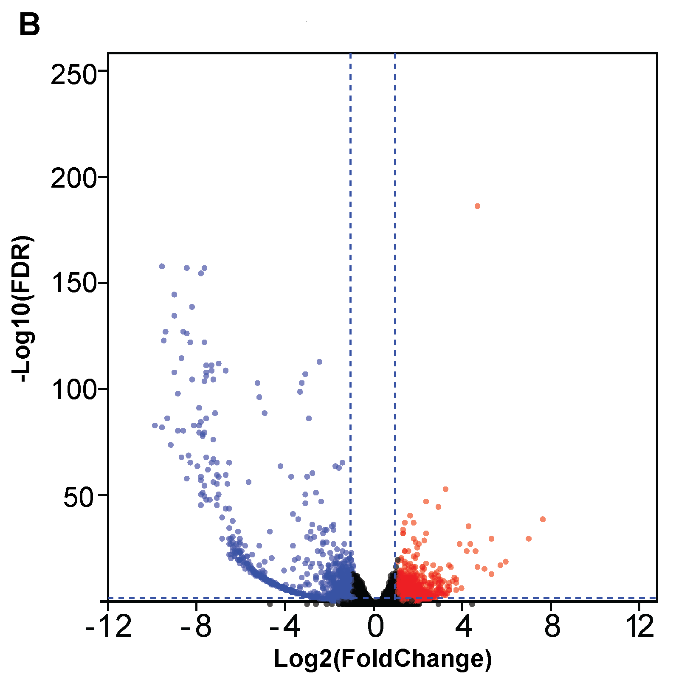

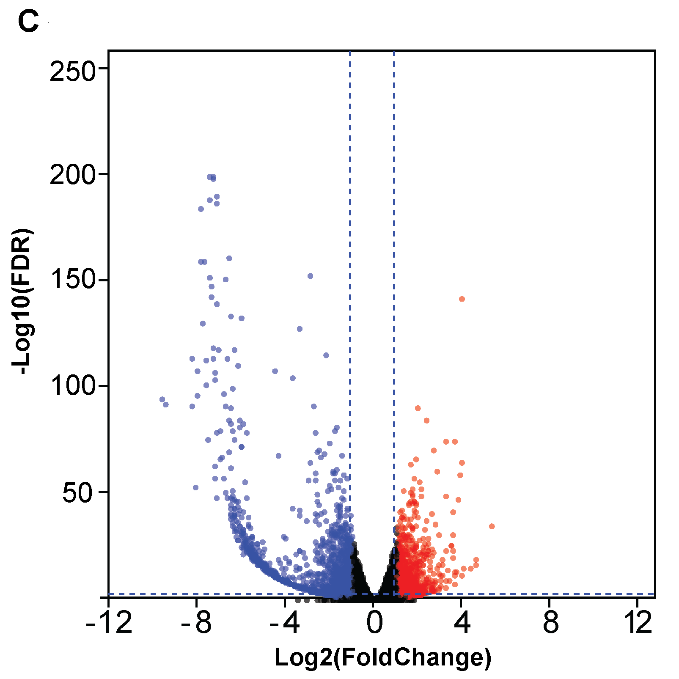

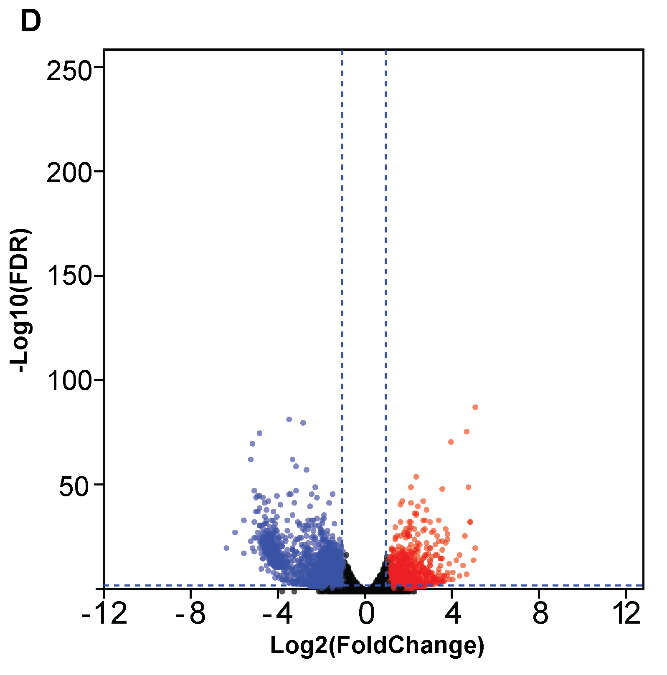
**

**Figure S2.** Volcano plots of the differentially expressed genes (DEGs) between control and treated *Myriophyllum aquaticum*. The abscissa represents changes of gene expression. The ordinate represents the statistical significance of change of the amount of gene expression. The less false discovery rate (FDR), the more −log10(FDR), and the more significance. The scattering dots represent genes, while the black dots show genes without significant differences, whilst the red and blue dots indicated significantly upregulated and downregulated genes, respectively. (a) L1 vs LNC. (b) L2 vs LNC. (c) L3 vs LNC. (d) L4 vs LNC. LNC: group treated with 0 mM ammonium; L1: group treated with 0.1 mM ammonium; L2: group treated with 1 mM ammonium; L3: group treated with 12 mM ammonium; L4: group treated with 36 mM ammonium.

**
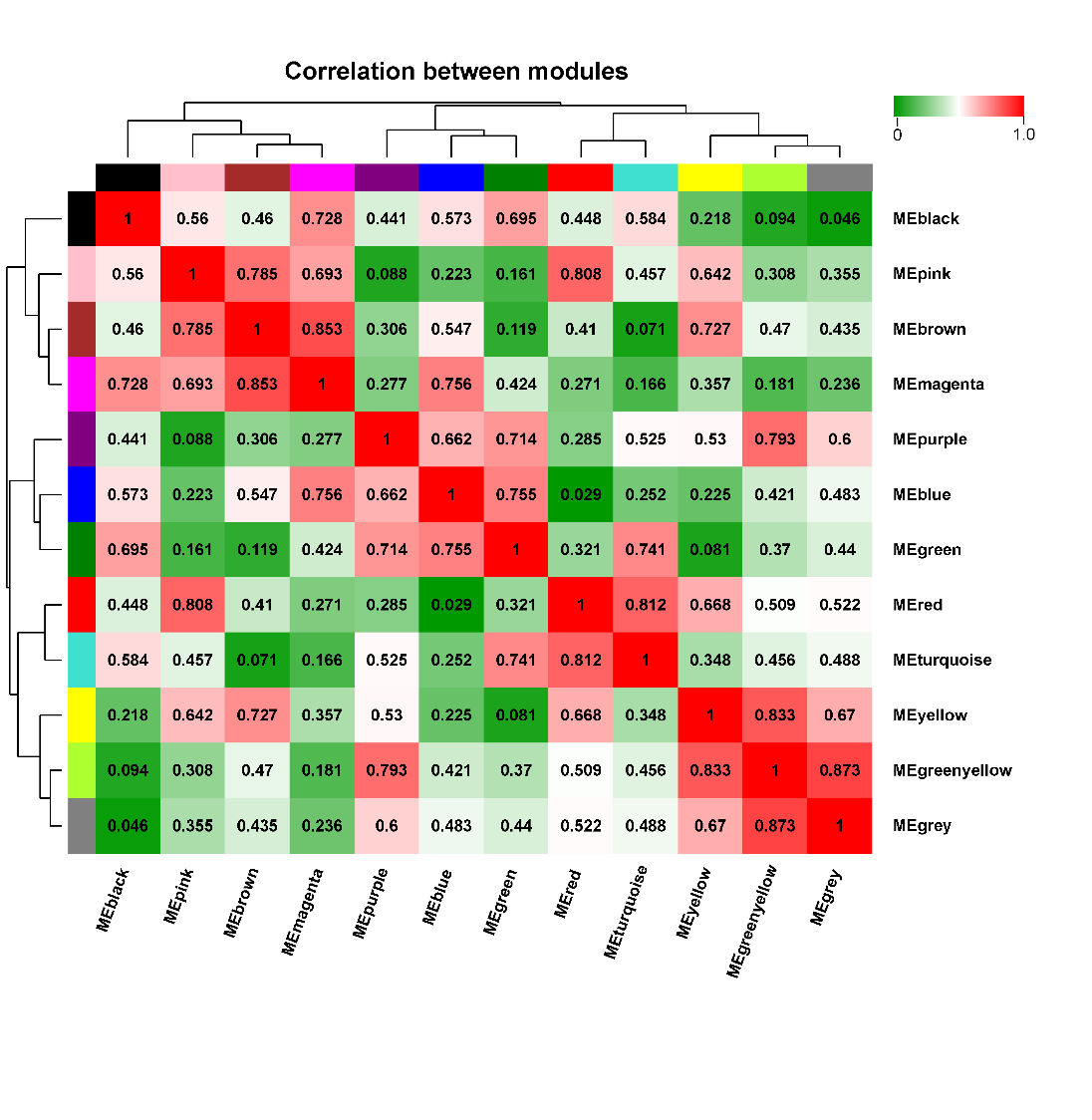
**

**Figure S3.** Heatmap of correlation analysis among 10 modules. Different colours on the top and left represent different modules. A correlation coefficient ranged from 0 to 1.


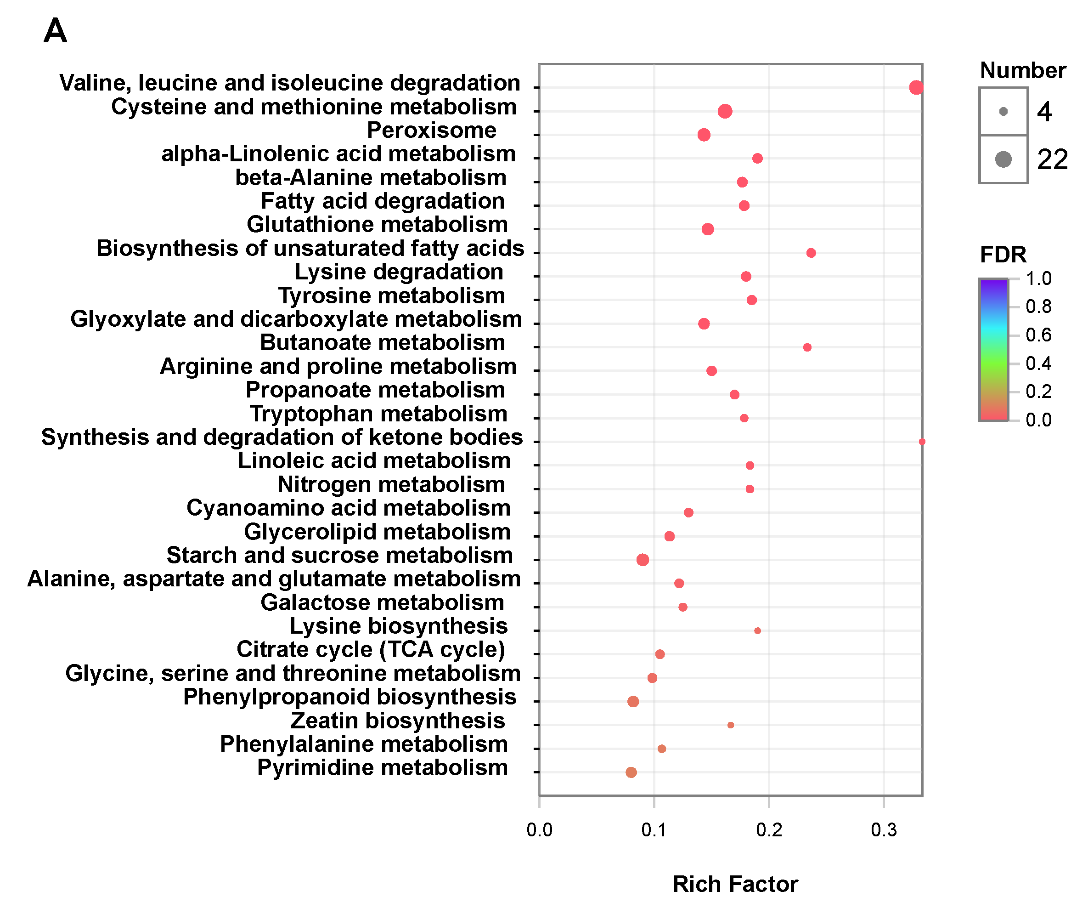

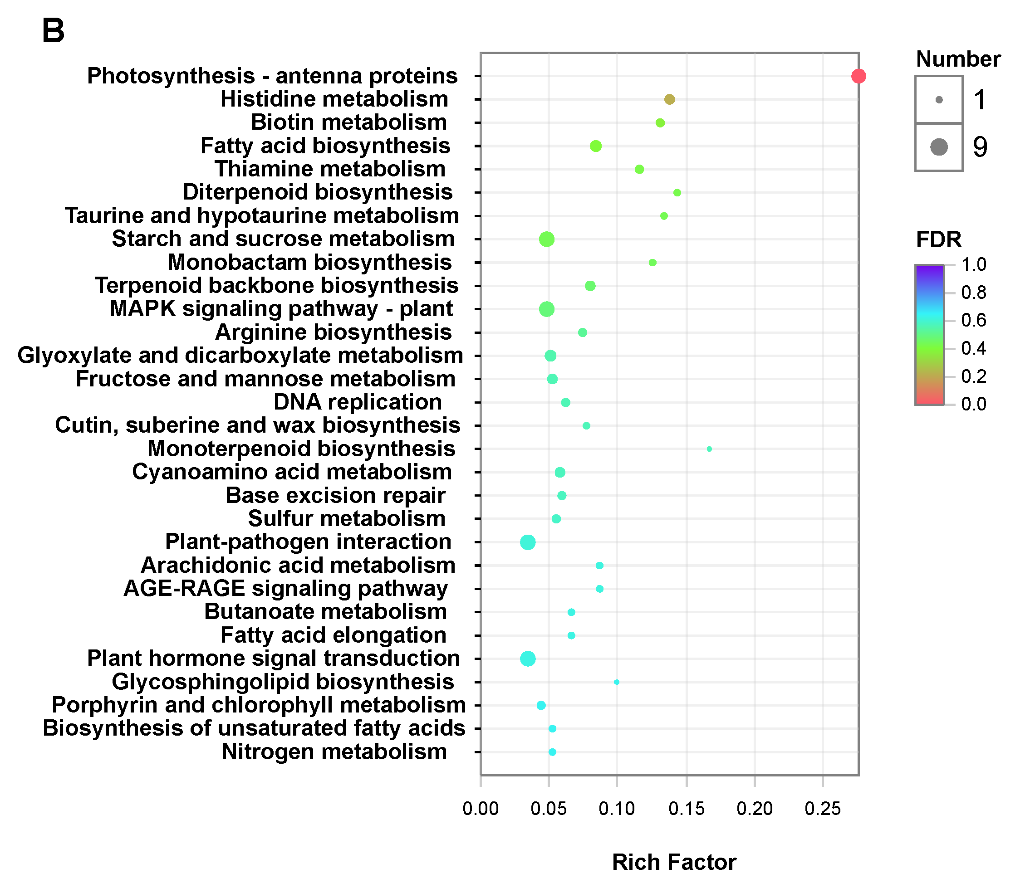


**Figure S4.** Scatterplot of top 30 Kyoto Encyclopedia of Genes and Genomes (KEGG) pathway enrichment for differentially expressed genes (DEGs) in the positive and negative modules.


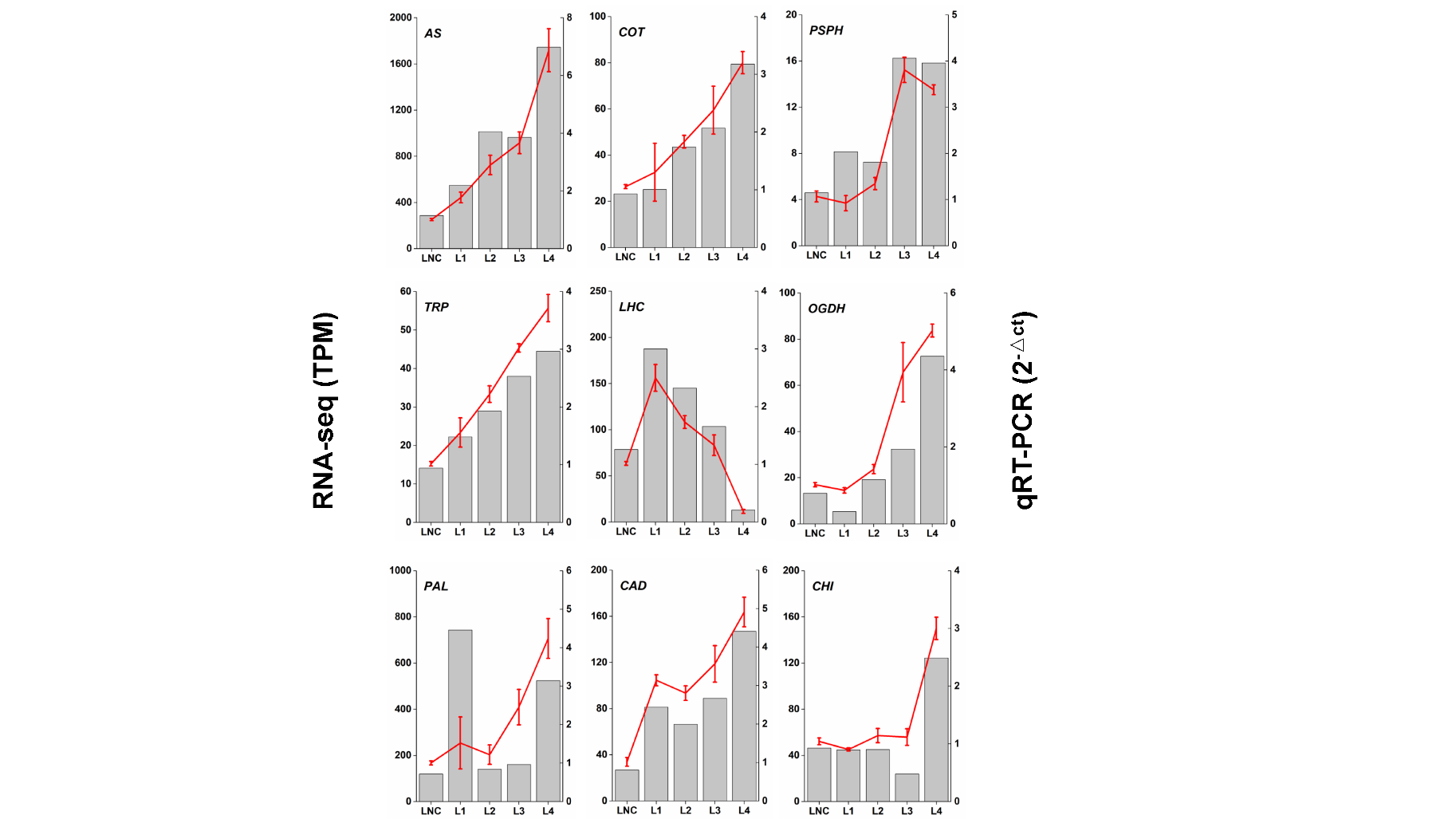


**Figure S5.** qRT-PCR validation of differentially expressed genes in *Myriophyllum aquaticum* at different concentrations of ammonium. The axes represent the transcripts per million reads (TPM) observed in RNA-seq analysis (grey bars) and the fold change in expression (red lines) observed in qRT-PCR analyses. LNC: group treated with 0 mM ammonium; L1: group treated with 0.1 mM ammonium; L2: group treated with 1 mM ammonium; L3: group treated with 12 mM ammonium; L4: group treated with 36 mM ammonium. Values (means ± SDs) were determined from three biological replicates.
